# Rapid Adaptation to Road Salts in a Freshwater Microbial Eukaryote

**DOI:** 10.1002/ece3.73160

**Published:** 2026-02-25

**Authors:** Rebecca A. Zufall, Nia Pereda, Karissa Plum, Ethan Rothschild

**Affiliations:** ^1^ Department of Biology and Biochemistry University of Houston Houston Texas USA

**Keywords:** adaptation, ciliate, experimental evolution, salinity, *Tetrahymena*, tradeoff

## Abstract

Humans are changing habitat for wildlife in myriad ways and for populations to persist, they must adapt to this change. In parts of the world that experience snow and ice, road salts are often used to make driving safer in the winter. Runoff from these roads increases the salinity in nearby bodies of water, which has been shown to have detrimental physiological and ecological effects in freshwater ecosystems; however, the evolutionary consequences of salinization remain unclear. *Tetrahymena* are microbial eukaryotes that live in freshwater habitats and serve as an important link in the microbial food loop. In this study, we tested how *T. thermophila* can evolve in response to increasing concentrations of road salts in their environment. Using experimental evolution, we found that *T. thermophila* populations adapted to survive and grow in concentrations of up to 18 g/L of NaCl and 17 g/L MgCl_2_, approximately twice the salinity tolerance of ancestral populations. However, populations adapted to the highest salt concentrations experience fitness trade‐offs of reduced survival and growth rate and longer lag times when grown in salt‐free environments. These results demonstrate the rapidity with which microbial populations can respond to anthropogenic changes to their environment, yet highlight the potential costs associated with this adaptation.

## Graphical abstract

Road salt application is causing increased salinization of freshwater ecosystems. Using experimental evolution, we demonstrate that ciliates can quickly adapt to tolerate high salinities, but this adaptation may come at a cost to survival in the absence of salt.

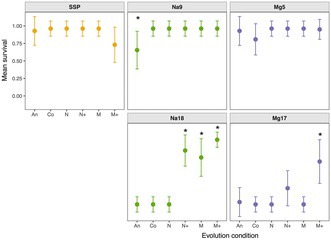

## 1 Introduction

Human activity causes a variety of types of disturbance resulting in novel environmental conditions that pose challenges to the organisms in that environment. If and how organismal populations will adapt to such rapid environmental change is an open question with important implications for biodiversity and ecosystem services (Peters et al. 2013; Reid et al. 2019). One important source of human‐caused environmental disturbance comes from the use of road deicing salts in regions of the world that experience cold winters. Application of road salts improves road safety but has led to dramatically increased salinization of freshwater ecosystems, particularly those adjacent to or experiencing runoff from heavily salted roads (Dugan and Arnott 2023; Hintz et al. 2022). In North America, the use of road salts started in the 1940's and has been increasing each year since then (Corsi et al. 2010; Kaushal et al. 2005). Sodium chloride is the most commonly used road salt worldwide, with magnesium chloride the second most commonly used in North America (Schuler et al. 2017). The effects of increased salinity in freshwater ecosystems include acute and chronic toxicity, changes in population dynamics, and reduced biodiversity (Corsi et al. 2010; Kaushal et al. 2005; Searle et al. 2016; Szklarek et al. 2022).

Several studies have examined the immediate impact of road salts on individuals, populations, and communities, demonstrating the potential for natural selection on salt tolerance (e.g., Hopkins et al. 2013; Schuler et al. 2017; Searle et al. 2016). Fewer studies have directly tested whether freshwater populations will adapt to increased salinity. Coldsnow et al. (2017) and Hintz et al. (2018) demonstrated that *Daphnia* populations rapidly evolve increased tolerance to road salts. Evidence of local adaptation in road‐adjacent populations of salamanders (Brady 2012) is also consistent with evolved tolerance to road salts. In contrast, Huber et al. (2024) found no increase in fitness in *Daphnia pulex* following evolution in salt. To further elucidate the long‐term consequences of salinization of freshwater ecosystems due to road salts, it is necessary to better understand the potential for populations of various organisms to evolve in these novel conditions.

Here, we studied the effects of road salts on the ciliate *Tetrahymena thermophila*. *T. thermophila* is a freshwater eukaryotic microbe endemic to ponds and streams in the northeastern United States (Zufall et al. 2013). Road salt is frequently applied in this region in the winter, so populations of *T. thermophila* near roads are likely experiencing increases in salinity levels. *T. thermophila* , like many ciliates, are a critical link in the microbial food loop, facilitating energy flow from bacteria to larger organisms (Esteban and Fenchel 2021; Weisse 2017). *Tetrahymena* are primary predators that feed largely on bacteria and are preyed upon by zooplankton and small vertebrates (D. Lynn 2008). Thus, to understand how freshwater food webs will respond to salinization, it is important to determine how this critical link will respond. Using a laboratory experiment, we tested how *T. thermophila* populations might respond to increasing salinity levels.

While some aspects of ciliate biology have been widely studied, less is known about their potential to respond to rapid environmental change. Ciliates are abundant and inhabit nearly every ecosystem worldwide (D. H. Lynn 2012), suggesting that they have the potential to adapt to novel environments. Ciliates are also a powerful system for experimental evolution due to their short generation time, ability to grow axenically in the lab, and available genetic tools (Plum et al. 2022). Using experimental evolution, we assessed the effects of road salts on populations of *T. thermophila* . We ask (1) whether and how rapidly populations can adapt to tolerate increasing salt concentrations, (2) whether there are tradeoffs for salt‐adapted populations in salt‐free environments, and (3) whether adaptation to one salt increases fitness in other types of salt.

## 2 Materials and Methods

### 2.1 Study System and Culture Conditions

*Tetrahymena thermophila* strain SB210E (*Tetrahymena* Stock Center ID SD01539), a highly inbred strain originally isolated from Eel Pond, a tidal pond in Woods Hole, MA in the 1950s (Doerder and Brunk 2012), was used for all experiments. This strain was chosen because it is a standard lab strain with many genetic resources available. Cells were grown in a standard nutrient‐rich *Tetrahymena* medium, SSP (2% proteose peptone, 0.1% yeast extract, 0.2% glucose, 0.003% Fe‐EDTA), which contains no chloride‐based salts, with added penicillin–streptomycin–amphotericin solution (Cassidy‐Hanley 2012; Gorovsky et al. 1975) for all experiments, either with or without added salts as described below. 100% SSP was used for growth conditions and 5% SSP was used for population maintenance with minimal cell division.

**FIGURE 1:**
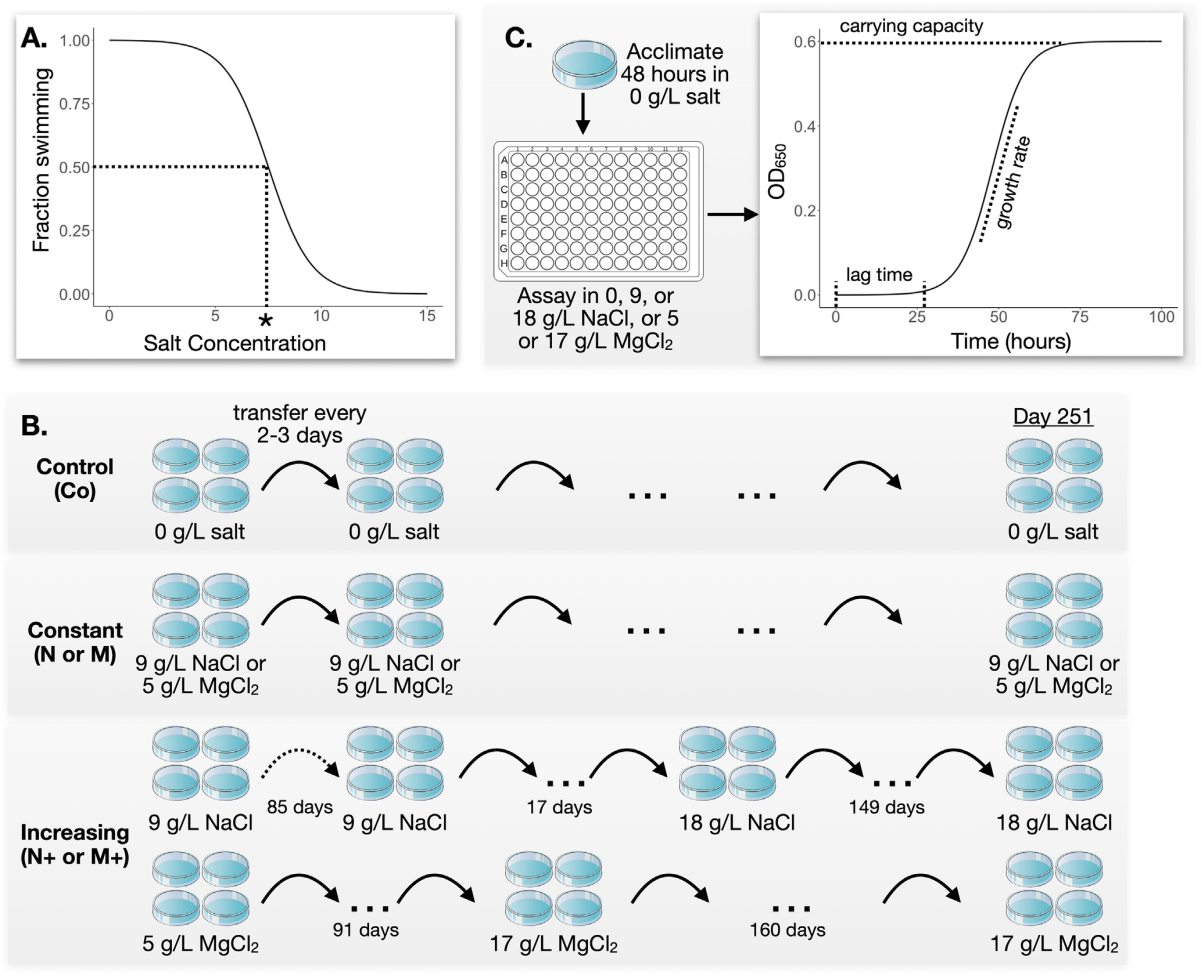
Experimental design. (A) Assay to determine starting salt concentrations. Ancestral *T. thermophila* were assayed for swimming and division at various salt concentrations. The inferred concentration at which 50% of cells were swimming or dividing, shown here with hypothetical data, is marked with an asterisk (*). This concentration (9 g/L NaCl or 5 g/L MgCl_2_) was used as the initial salt concentration in the evolution experiment. (B) Design of evolution experiment. Populations of *T. thermophila* were grown in three different types of conditions, all of which were subcultured to new medium every 2 or 3 days, with 4 replicate populations for each treatment. “Control” (Co) populations were grown with no salt (0 g/L). “Constant” populations were grown in a constant concentration of salt for the duration of the experiment: 9 g/L of NaCl (N) or 5 g/L of MgCl_2_ (M). Control and constant populations were maintained in the same conditions for 251 days. “Increasing” populations were grown in increasing concentrations of salt until they could no longer adapt to the higher concentration. For the increasing lines in MgCl_2_ (M+) the concentration of salt was increased by 10% each week for the first 91 days, until reaching a concentration of 17 g/L. These populations were then maintained at this concentration for another 160 days. The increasing populations in NaCl (N+) experienced a more complicated regime due to the loss of populations during the experiment. Backup populations of the N+ lines were maintained in 9 g/L NaCl and subcultured under the same regime as the constant N lines. After 85 days, the original N+ lines were lost and new N+ lines were started from 4 independent backup lines that had been in 9 g/L NaCl for 85 days (indicated by the dashed arrow). Over the next 17 days, NaCl concentration was increased by 3 g/L every ~6 days. The concentration was then maintained at 18 g/L for another 149 days. (C) Salt tolerance assays. Prior to assaying salt tolerance, all lines were first acclimated by growing for 48 h in 0 g/L salt to reduce the physiological effects of their previous environment. Cells were then moved to a 96‐well assay plate containing one of the 5 salt concentrations. All lines were assayed in all salt conditions. Changes in density due to cell division were monitored by measuring OD_650_ on a microplate reader. Growth curves were plotted to extract values corresponding to the three phases of microbial growth: time in lag phase, growth rate during the exponential phase, and maximum density representing carrying capacity during stationary phase.

Prior to the start of the experiment, *T. thermophila* was assayed for salt tolerance to determine the appropriate starting concentrations of salt for experimental evolution (Figure 1A). 200 μL of cells were put into replicate wells with 0, 3, 6, 9, 12, or 15 g/L of NaCl or MgCl_2_ in SSP. 24 h following salt exposure, cells were observed under a microscope for movement and cell division. Dose response curves were plotted as the salt concentration vs. fraction of cells swimming or dividing. The salt concentrations at which ~50% of cells were not swimming or dividing were used as the starting concentrations for experimental evolution. This allowed us to impose strong selective pressure while maintaining sufficient levels of cell division.

### 2.2 Experimental Evolution

Prior to the start of the experiment, *T. thermophila* were thawed from liquid nitrogen storage and a single cell was isolated and grown to stationary phase for 1 week in SSP at 24°C. 300 μL of cells were transferred to wells of a 12‐well plate, in 3.5 mL of SSP, containing either no added salt (this was the control for adaptation to the laboratory conditions), 9 g/L NaCl, or 5 g/L MgCl_2_ (Figure 1B). The salt treatments were either “constant,” that is, the same concentration of salt was used throughout the experiment (designated N and M for constant NaCl or MgCl_2_, respectively), or “increasing,” that is, the salt concentration was increased each week of the experiment until the populations could no longer adapt (designated N+ and M+; Table 1; Figure 1B). Each experimental treatment had four replicate populations and all populations were grown at 24°C in an incubator.

**TABLE 1:** Experimental conditions for each treatment.

Abbreviation	Description	Number of replicate populations	Total generations^a^	Time to reach maximum salinity^b^
An	Ancestor	1	~0	n/a
Co	Control: Evolved in 0 g/L salt	4	~542	n/a
N	Evolved in constant 9 g/L NaCl	4	~241	n/a
N+	Evolved in increasing NaCl up to 18 g/L	4	~662	102 days
M	Evolved in constant 5 g/L MgCl_2_	4	~783	n/a
M+	Evolved in increasing MgCl_2_ up to 17 g/L	4^c^	~120	91 days

^a^ Total number of generations is roughly estimated based on the average maximum population growth rate measured in growth assays in the evolved condition (Figure 4) multiplied by 251 days. For N+ and M+, growth rates in their final concentration were used even though not all evolution took place at that concentration.

^b^ Number of days of evolution before N+ and M+ reached their final salt concentrations.

^c^ One population was lost prior to completing all growth assays.

Each population was subcultured to a new well every 48–72 h. Cells were thoroughly mixed via pipetting, then 10% was transferred to fresh medium for a final volume of 3.5 mL. At each transfer, cultures were visualized under the microscope to ensure ongoing cell division and no contamination. When contaminants (usually fungal) were observed, new subcultures were started from the most recent prior culture of that replicate that did not have a contaminant.

The N+ lines reached a concentration of 21 g/L NaCl after 62 days, but then all populations died. These populations were restarted from backup populations that had been adapting to 9 g/L NaCl for 85 days, then took an additional 17 days to reach 18 g/L NaCl (Figure 1B). The NaCl concentration was not increased beyond 18 g/L so cell lines would not be lost again (Note that the original N+ populations reached 18 g/L NaCl after 50 days). Thus for NaCl, the final concentration in the increasing populations was 18 g/L. For MgCl_2_, the final concentration was 17 g/L, reached after 91 days.

Populations were maintained in their salt treatments for a total of 251 days (Figure 1B), or approximately 500 generations on average. Cell counts were not made during the experiment, so we estimated the elapsed number of generations based on the average maximum population growth rate of each population in its evolved condition (Table 1).

The ancestor that was used in the growth assays at the end of the experiment was maintained in a 5% solution of SSP medium throughout the 251 days. This allowed cells to survive for extended periods of time with little cell division and thus little opportunity for evolution. Once the evolution experiment was completed, all populations were moved to 5% SSP (plus the same/final concentration of salt as their experimental condition) or liquid nitrogen storage (Cassidy‐Hanley et al. 1995) until assays could be performed.

### 2.3 Growth Assays

Following experimental evolution, all populations were assayed for growth in each salt condition. Prior to starting a growth assay, all cells were grown in SSP with no salt for at least 2 days (Figure 1C). This period of acclimation ensures that any differences observed between lines are due to evolved differences, rather than plastic responses to their growth conditions prior to starting the assay. Following acclimation, cell density was determined by counting under a microscope. Approximately 150 cells were transferred to wells in a 96‐well plate containing 170 μL of medium. Five assay plates were used containing the salt concentrations used in each experimental condition, or the final concentration for the increasing treatments: no salt (SSP), 9 g/L NaCl (Na9), 18 g/L NaCl (Na18), 5 g/L MgCl_2_ (Mg5), and 17 g/L MgCl_2_ (Mg17). Cells from each of the experimental populations, and the ancestor, were randomized on each plate. Each population was inoculated into 3 wells each (for triplicate measurement replication), except the ancestor, which had 12 replicates. The remaining wells contained media. Prior to completing the two MgCl_2_ assays, one of the M+ populations was lost. To monitor changes in cell density, OD_650_ was measured on a microplate reader every 10 min, with shaking, for 6 days. Following completion of the assay, all wells were inspected microscopically to ensure no contamination and assess which wells had living cells.

### 2.4 Data Analysis

#### 2.4.1 Growth Curve Fitting

Growth curves generated with the OD_650_ versus time data from growth assays (Figure 1C) were analyzed using the R package growthrates (Petzoldt 2022). Because we observed that lag time varies greatly among treatments, we only fit growth models that contained a parameter for lag time. The Huang (2011) model provided the best overall fit based on residual sum of squares. Three parameters were extracted from the fit curves using the Huang model implemented in growthrates: maximum growth rate, carrying capacity, and lag time.

#### 2.4.2 Survival Analysis

Growth curve models that fit the data with *r* ^2^ > 0.90 were considered to have survived. Visual inspection of growth model fits confirmed that models with *r* ^2^ < 0.90 did not fit well because OD values never increased above the detectable threshold, indicating little or no cell division in that well due to cell death. Cell death in these conditions was confirmed by staining with the vital dye trypan blue.

Survival probability was analyzed with a logistic model to test for effects of experimental condition, that is, salt concentration during the experiment, and assay condition, that is, salt concentration during the growth assays (survival ~ experimental condition × assay condition). Separation in the data was detected using maximum likelihood estimates of parameters with the R package detectseparation (Kosmidis et al. 2022). Due to the complete separation of the data, we used brglm_fit to implement mean bias reduction (Kosmidis et al. 2020). Marginal means and contrasts were found using the R package modelbased (Makowski et al. 2025).

#### 2.4.3 Dose Response Curves

For dose response curves, we combined survival data from the two salts and considered only chloride ion concentration (survival probability ~ [Cl^−^]). The R package drc (Ritz et al. 2015) was used to fit dose response curves, extract IC50 values, and test for pairwise differences in IC50 values.

#### 2.4.4 Growth Parameter Analysis

For assayed populations that survived, we further analyzed growth curve parameters from the model fit growth curves. Populations that did not reach carrying capacity during the assay (visually identified as wells with carrying capacity > 0.8 or maximum growth rate > 0.25) were removed from the analysis. Linear mixed effect models were used to assess the effects of experimental condition and assay condition on the three growth parameters: carrying capacity, maximum growth rate, and lag time (growth parameter ~experimental condition × assay condition + replicate population nested within experimental condition) using the R package lmerTest with restricted maximum likelihood (Kuznetsova et al. 2017). Replicate population is a random effect and nested within experimental condition; experimental and assay conditions are fixed effects. The R package emmeans was used to estimate marginal means and perform pairwise comparisons (Lenth and Piaskowski 2025).

## 3 Results

Replicate populations of *T. thermophila* were cultured under either constant or increasing concentrations of two of the most commonly used road salts, NaCl and MgCl_2_. After ~500 generations, changes in salt tolerance of all populations were measured via population growth curves, which were used to estimate survival probability and growth parameters (Figure 1).

### 3.1 Survival of Evolved Populations

**FIGURE 2:**
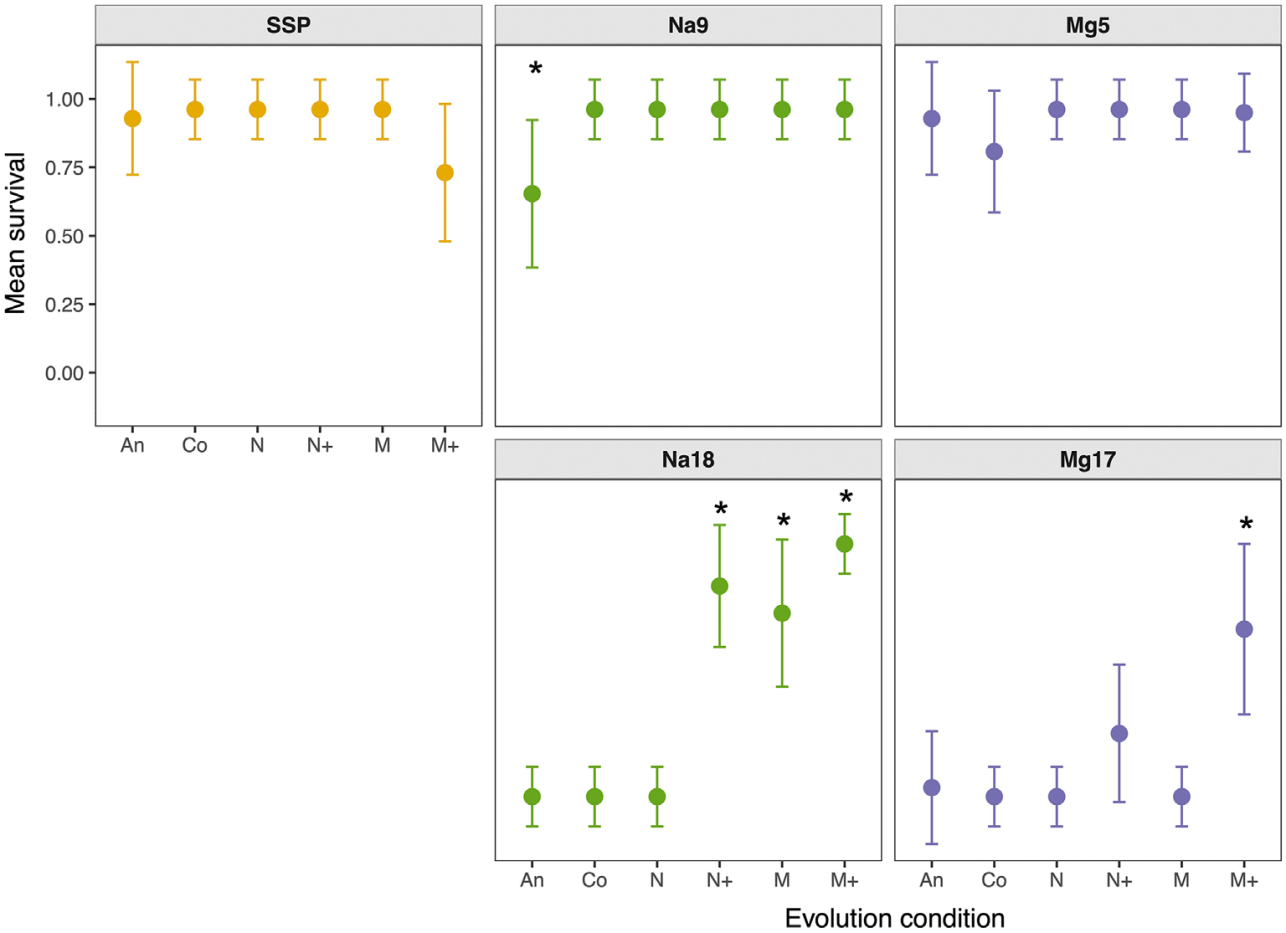
Survival of each population in no salt (SSP), medium salt: 9 g/L NaCl (Na9) or 5 g/L MgCl_2_ (Mg5), or high salt: 18 g/L NaCl (Na18) or 17 g/L MgCl_2_ (Mg17). Survival is the marginal mean of the replicate populations shown with 95% confidence intervals. Experimental conditions are as in Table 1. Asterisk (*) indicates values that differ significantly from the others in marginal contrasts within assay condition (*p* < 0.05), except that M+ is not significantly different from N+ in Mg17 (Table S1).

Survival was defined by the ability of cells to survive and divide in each salt concentration. Experimental condition (χ^2^_5_ = 32.25, *p* < 0.001), assay condition (χ^2^_4_ = 234.94, *p* < 0.001), and the interaction between these (χ^2^_20_ = 37.94, *p* = 0.009) all significantly affected the probability of survival. Survival was high for all populations in the no‐salt (SSP) and medium‐salt conditions (Na9 and Mg5), with two exceptions (Figure 2). The ancestor had decreased survival in 9 g/L NaCl relative to the other populations (Δ = 0.31, *p* = 0.038; Table S1). In addition, the M+ populations had a decrease in survival in the no‐salt condition relative to the other populations, though this was not significant (Δ = 0.20–0.23, *p* > 0.05; Table S1).

In high‐salt conditions (Na18 and Mg17), the increasing‐salt evolved lines tended to survive much better than the others (Figure 2). In the 18 g/L NaCl assay condition, M, M+, and N+ populations all survived significantly better than the ancestor and control populations (Figure 2; Δ = 0.67–0.92, *p* < 0.001; Table S1). In 17 g/L MgCl_2_, M+ survived better than all other lines, except N+ (Figure 2; Δ = 0.58–0.61, *p* < 0.01; Table S1).

### 3.2 Dose Response Curves

**FIGURE 3:**
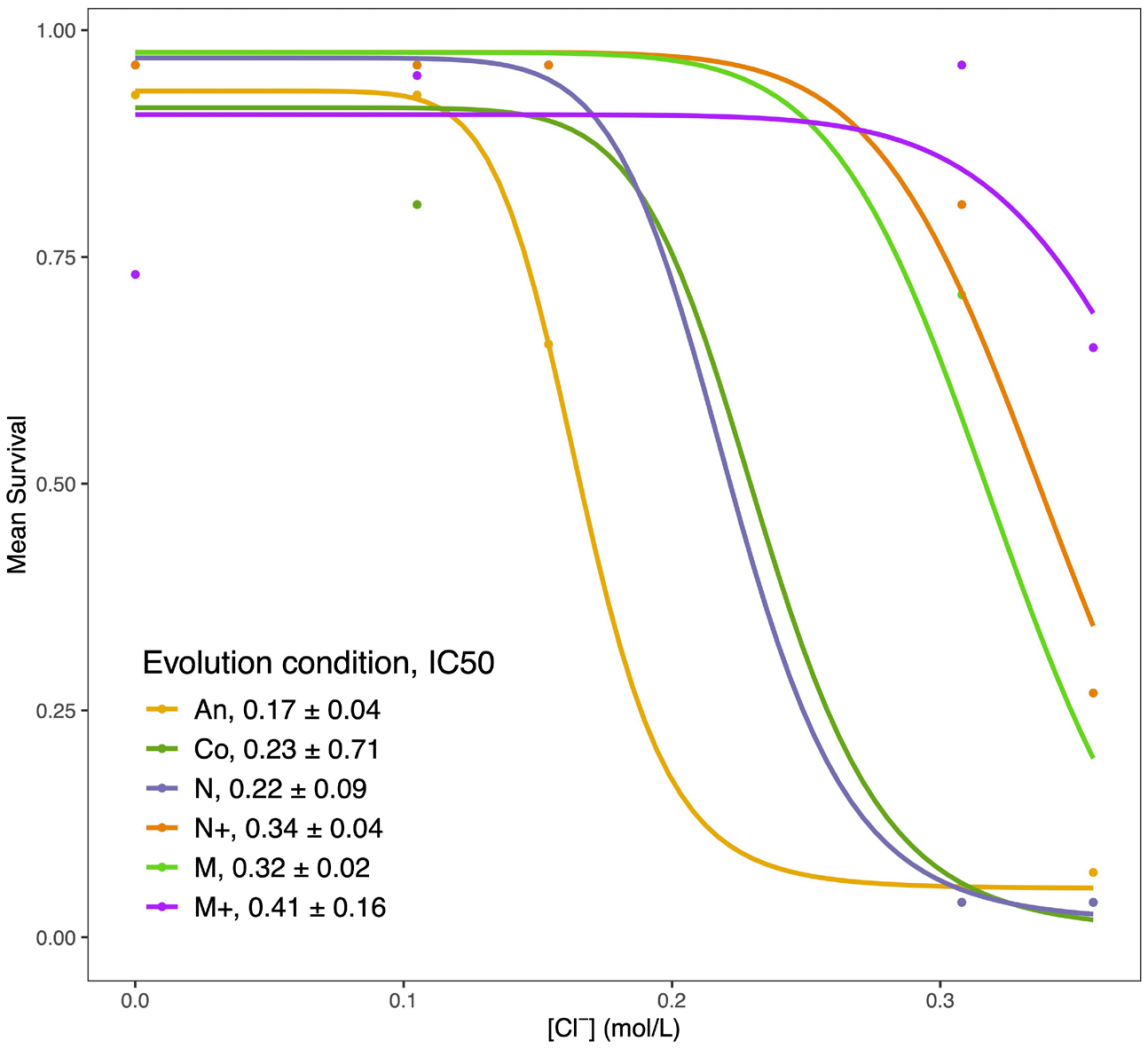
Dose response curves based on marginal means of survival probability in different chloride ion concentrations. Curves were fit using a four‐parameter log‐logistic function in the R package drc. IC50 values are shown numerically ± standard error. Colored points are the marginal means for populations from each experimental condition.

Combining the data from NaCl and MgCl_2_ assays, we determined half‐maximal inhibitory concentrations (IC50) of chloride using dose response curves (Figure 3). IC50 corresponds to the chloride concentration at which half of all replicate populations are expected to not survive at that concentration. All evolved lines, including the control, had IC50 values trending higher than the ancestor. IC50 was significantly higher than the ancestor in N+ and M lines (*t*‐test, *p* < 0.05). The estimate of IC50 is highest in the M+ line; however, this estimate has a high degree of uncertainty because it had high survival rates even in the highest measured salt concentrations (Figure 3).

### 3.3 Effect of Salt Adaptation on Growth Parameters

Three growth parameters were estimated. (1) Lag time, defined as the delay before exponential growth, is affected by the rate at which cells acclimate to the growth environment. The temporal dynamics of this phenotypic plasticity have been shown to be important to adaptive evolution (Dupont et al. 2025). (2) Maximum growth rate during exponential phase represents the maximum rate of asexual reproduction. (3) Carrying capacity at stationary phase, the maximum density reached in a well, represents the maximum number of cells that can be supported in the population. All three growth parameters were significantly affected by experimental condition (lag time: *F*_5,13.74_ = 29.24, *p* < 0.001; growth rate: *F*_5_,_12.06_ = 22.08, *p* < 0.001; carrying capacity: *F*_5_,_14.07_ = 29.07, *p* < 0.001), assay condition (lag time: *F*_4_,_200.06_ = 593.96, *p* < 0.001; growth rate: *F*_4_,_191.87_ = 97.74, *p* < 0.001; carrying capacity: *F*_4_,_176.20_ = 105.57, *p* < 0.001), and their interaction (lag time: *F*_13,197.90_ = 43.61, *p* < 0.001; growth rate: *F*_13_,_190.00_ = 28.78, *p* < 0.001; carrying capacity: *F*_12_,_174.38_ = 28.46, *p* < 0.001) indicating evolved differences in response to environmental change.

**FIGURE 4:**
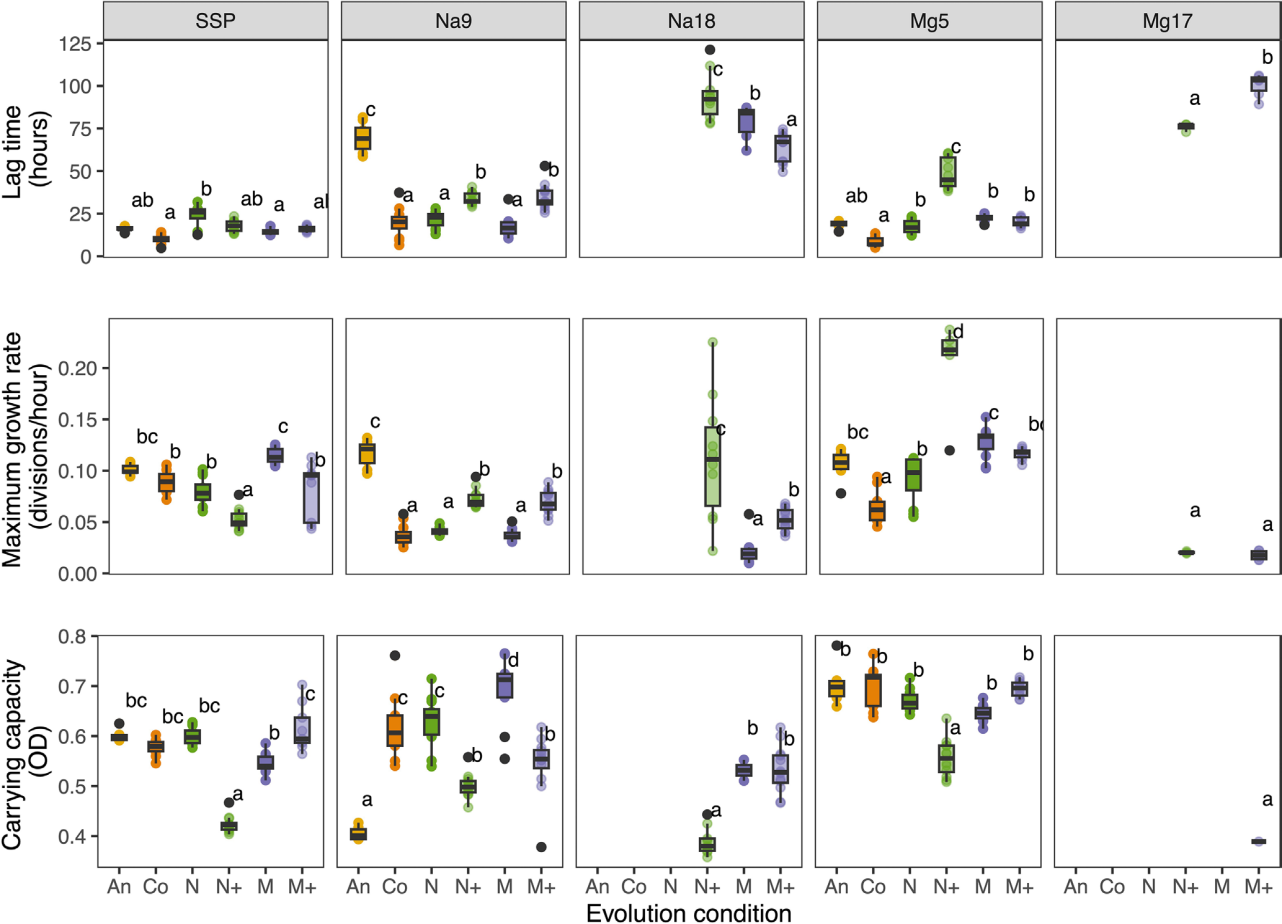
Growth parameters of surviving populations. Lag time, maximum population growth rate, and carrying capacity were estimated from growth curve models of each population assayed in each salt concentration. Abbreviations are as in Figure 2. Pairwise comparisons of marginal means within each assay condition are shown; values that share a letter are not significantly different from one another at *p* < 0.05.

The ancestor generally did not differ from most other lines when grown in 5 g/L MgCl_2_, but had a significantly longer lag time and lower carrying capacity than nearly all of the evolved lines in 9 g/L NaCl (all pairwise contrasts [except carrying capacity in An—N+], *p* < 0.001; Figure 4; Table S2). This is consistent with the fact that 5 g/L MgCl_2_ is below the IC50 of the ancestor and 9 g/L NaCl is approximately equal to the ancestor's IC50.

**FIGURE 5:**
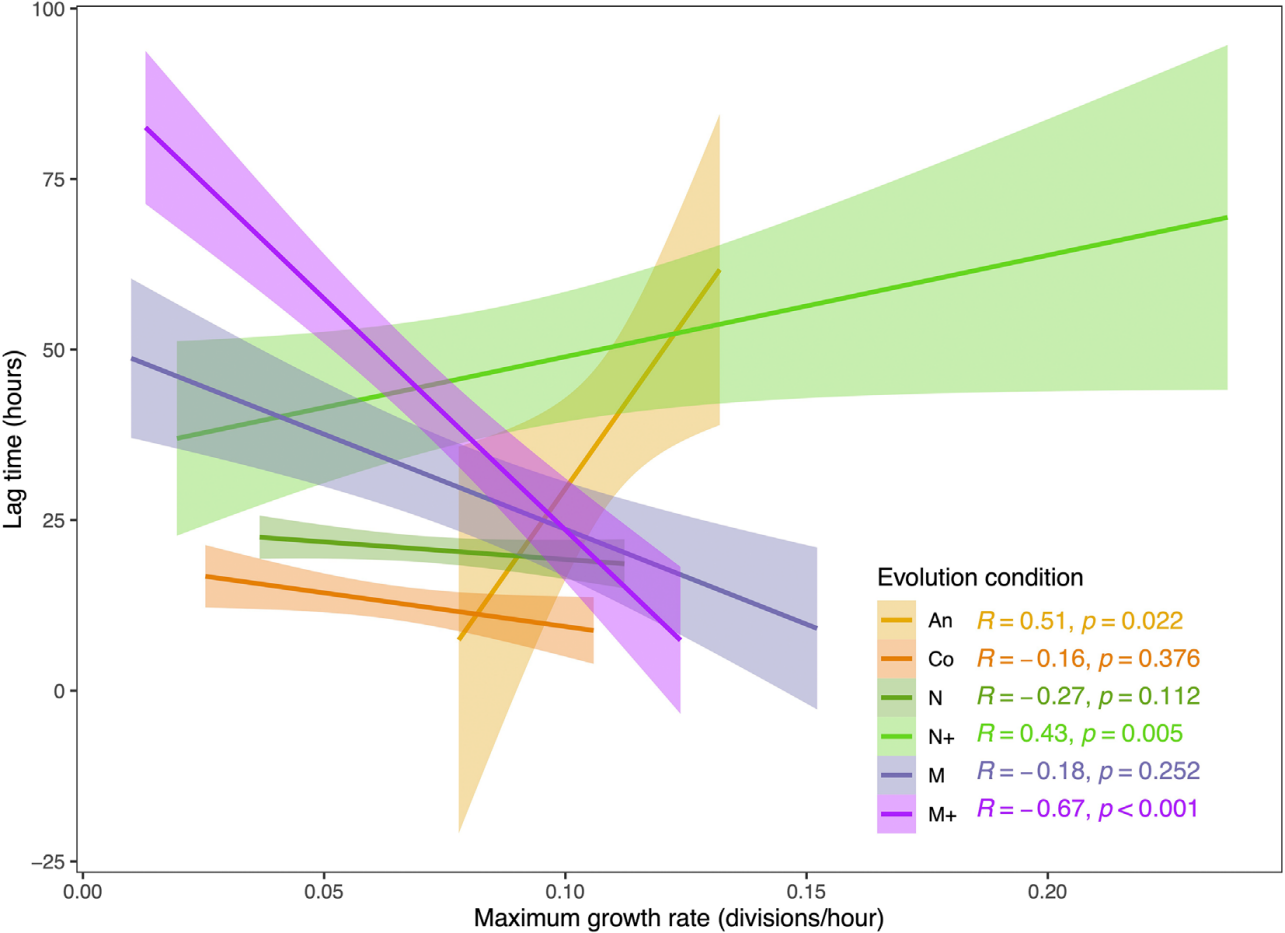
Correlation between lag time and growth rate differs between evolution treatments. Linear regression, 95% confidence interval, and Spearman correlation coefficient are shown for each experimental condition.

Surprisingly, the maximum growth rate of the ancestor in 9 g/L NaCl is significantly higher than the other populations (all pairwise contrasts *p* < 0.01; Figure 4; Table S2). The pattern of a long lag time and low carrying capacity in conjunction with a faster growth rate is also observed in the N+ populations when grown in the medium salt conditions and 18 g/L NaCl. Looking at all of the populations together, there is not a significant correlation between lag time and growth rate (Spearman rank correlation, *R* ^2^ = −0.084, *p* = 0.21), but the ancestor and N+ populations both show a positive correlation between these parameters whereas all other populations show a negative relationship (Figure 5).

The control populations, that is, those evolved in no added salts, outperformed the ancestor when grown in 9 g/L NaCl, with shorter lag times (estimate = 49.68 ± 3.28, *t*_29.9_ = 15.16, *p* < 0.001) and higher carrying capacity (estimate = 0.209 ± 0.026, *t*_22.1_ = 8.17, *p* < 0.001; Figure 4; Table S2). They also survived slightly better and had higher IC50s than the ancestor (Figures 2 and 3).

N+ and M+ populations evolved the highest salt tolerances, but this came with a cost when grown in no‐salt or medium‐salt concentrations. In particular, N+ populations had significantly reduced growth rates (all pairwise contrasts *p* < 0.05) and carrying capacities (all pairwise contrasts *p* < 0.001) compared to all other populations when grown in no salt (SSP), and longer lag times and lower carrying capacities than the control, N, and M evolved populations when grown in medium‐salt concentrations (Figure 4; Table S2). M+ populations only show increased lag times and reduced carrying capacity in 9 g/L NaCl relative to the control, N, and M populations. In addition, slightly fewer M+ populations survived in SSP (Figure 2), however, those that survived exhibited growth patterns in SSP similar to the other populations (except N+; Figure 4; Table S2).

## 4 Discussion

Freshwater ecosystems are experiencing detrimental effects of increased salinization due to application of road salts (Dugan and Arnott 2023; Hintz et al. 2022). Determining how populations will adapt to this environmental challenge is critical for understanding the future health of these habitats. Here, we demonstrated that *T. thermophila* has the potential to adapt quickly to increasing concentrations of two of the most commonly used road salts. Following evolution in salt, ciliate populations were able to survive in significantly higher salt concentrations than the ancestral population. This result is consistent with previous studies in *Daphnia*, which were also shown to quickly evolve increased tolerance to road salts (Coldsnow et al. 2017).

Considering these results in the context of wild populations, measured concentrations of Cl^−^ in freshwater systems in the northeastern United States have been reported to reach as high as 0.03–0.13 mol/L (Brady and Benoit 2025; Huber et al. 2024; Kaushal et al. 2005), close to the IC50 of the *T. thermophila* ancestral population. This suggests that natural populations of ciliates are likely to experience similar selective conditions as those imposed by our Na9 (0.154 mol/L) and Mg5 (0.105 mol/L) treatments. In addition, the timescale of our experiment is short enough that there would be sufficient time in a single season to see the responses we observed in lab. In particular, the N+ and M+ lines adapted to survive in high salinity after only 50 (for the original N+ lines that were lost) or about 100 generations (Table 1). While we do not know how rapidly cells are dividing in natural conditions, under laboratory conditions they can reproduce every couple of hours, thus it is likely that they undergo more than 100 generations in a single year and may experience similar salinities to those in our experiment, suggesting that the responses observed here may be relevant to wild populations.

We are cautious, however, about applying our results based on a single lab strain to wild populations for many reasons. One point of caution comes from our unexpected result that lab adaptation in the control lines leads to increased salt tolerance even in the absence of salt in the environment. Interestingly, the lab strain used here was originally isolated ~70 years ago from a tidal pond, suggesting that the original isolate likely had high salt tolerance and may be naturally predisposed to quickly adapt to salt. Additionally, we recently found that other wild strains of *T. thermophila* also tend to have naturally high tolerance to NaCl, while another species, *T. ellioti* , did not (Swain et al. 2026), indicating substantial variation among ciliate species in salt tolerance. We did not consider here the effects of variation within or between populations that may also contribute to adaptation to novel environments. Testing the effects of high salt environments on other strains of *T. thermophila* and other ciliate species will further help to elucidate the issue of how populations will respond to increasing salinity.

Another factor from natural populations that was not taken into consideration in this experiment is the fact that application of road salts is not constant across time. Because road salts are applied in winter months, the highest salinity levels tend to be found in winter and early spring, with salinity decreasing through summer and fall (Brady and Benoit 2025; Dugan and Arnott 2023; Kaushal et al. 2005). These fluctuations in salinity levels are of particular interest given the observed fitness trade‐offs in the lines adapted to the highest salt concentrations. Compared to the freshwater adapted ancestor, N+ and M+ lines evolved the highest salt tolerance, but had reduced survival (M+), reduced growth rate (N+), and longer lag times (M+ and N+) when grown in salt‐free conditions. This suggests that adaptation to high salinity in spring can be costly when salinity is lower later in the year. Further experiments testing the effects of these annual fluctuations in salinity levels will be important to determine the long‐term effects of road salts on these populations.

Tradeoffs, whereby increased fitness in one environment leads to decreased fitness in another, such as those we find in the N+ and M+ lines, have been found in a wide variety of situations across taxonomic groups (e.g., Agudelo‐Romero et al. 2008; Gompert and Messina 2016). However, positive correlated responses, where selection in one environment leads to increased fitness in another, have also been found in a variety of contexts (e.g., Magalhães et al. 2009; Nidelet and Kaltz 2007; Olazcuaga et al. 2021; Tarkington and Zufall 2024). Here, we find positive correlated responses to selection whereby evolution in NaCl increases fitness in MgCl_2_ and vice versa. Given the similarity of these environments, perhaps this correlation is not surprising, although previous studies have found different responses to different road salts (Huber et al. 2024). More surprising is the fact that the control lines, evolved in no salt, also showed a positive correlated response when grown in salt, with an IC50 nearly the same as the lines evolved in 9 g/L NaCl. The mechanism for this response is unclear but corresponds to results from a previous study on experimentally evolved lines of *T. thermophila* , which showed frequent positive correlated responses to selection where evolution in one environment led to increased fitness in other environments (Tarkington and Zufall 2024). Here, it appears that adaptation to lab conditions has the correlated effect of increasing salt tolerance, though the mechanism is unknown.

Considering a different kind of tradeoff, Huber et al. (2024) found life history tradeoffs in *Daphnia* grown in salt. When grown in low salt, *Daphnia* had decreased brood size but increased lifetime reproductive output due to longer life spans. In the An and N+ populations here, we observe a similar life history tradeoff, where long lag times are followed by high growth rates. The usual expectation for the relationship between lag time and growth rate is a negative correlation—low fitness genotypes will have both long lag times and low growth rates. This has been found extensively (e.g., Baranyi and Roberts 1994; Basan et al. 2020) and is what we found in all of the populations besides An and N+. However, in the An and N+ populations, an inverse relationship indicates that these populations tend to take a longer time to acclimate to a new condition, but once they do, they are able to tolerate it better and grow faster. It is interesting that the two populations that show this pattern have the lowest and one of the highest salt tolerances.

One possible explanation for the observed tradeoffs could be due to selective differences among populations at different densities. We did not monitor density during the experiment; however, based on the observed differences in carrying capacity, it is likely that population densities differed between treatments. Population demography can alter selection and evolutionary trajectories. For example, at high densities, populations of *T. thermophila* evolved increased density‐dependent fitness via convergence of growth rate and intraspecific competitive ability (Moerman et al. 2020). Future experiments should explicitly consider population densities to test for density‐dependent effects of selection in novel environments.

Ciliate reproductive biology adds an additional complication to understanding how populations will adapt to increasing salinity. Ciliates contain two types of nuclei in each cell: a germline micronucleus, which is quiescent during asexual reproduction, and a somatic macronucleus, from which all gene expression is driven during growth and asexual reproduction (Merriam and Bruns 1988; Prescott 1994). In this experiment, all reproduction was asexual because we started with a single‐cell isolate that expresses only a single mating type and a different compatible mating type is required for sexual conjugation. Thus, the adaptation that we observed occurred solely due to mutations in the macronucleus. However, following sexual conjugation, the macronucleus gets degraded and a new macronucleus will develop from a zygotic nucleus (the product of syngamy between two haploid micronuclei; Orias et al. 2011). This means that any mutations that were selected in the macronucleus during asexual division will be lost following sex. Verdonck et al. (2022) point out that this ciliate lifestyle means that selection on somatic mutations is thus a form of phenotypic plasticity that will not have the same long‐term consequences as selection on germline mutations. It remains unknown when or how frequently *T. thermophila* undergo sexual conjugation in the wild (Doerder et al. 1995; Lynn and Doerder 2012; Zufall 2016), however, it is clear that this will change the dynamics of adaptation in these populations. Note that many *Tetrahymena*, including some lineages of *T. thermophila* , are obligately asexual, thus somatic adaptation is the only possible type of adaptation in these lineages (Doerder 2014). Further theory and experiments are necessary to understand the effects of sexual reproduction and generation of a new macronucleus for ciliate adaptation to increasing salinity and other types of environmental change.

Understanding the physiological and genetic mechanisms underlying the responses we observed would shed light into the mechanisms behind the observed tradeoffs and correlated responses to selection and the relationship between plastic and adaptive change, as well as the types of mutations responsible for adaptive evolution. Various cellular functions may be involved in maintaining osmotic homeostasis under saline conditions, such as ion transport and production of osmolytes (Kahle et al. 2015; Weinisch et al. 2019). Studies of halophilic ciliates demonstrate that they maintain homeostasis by eliminating excess salt ions and accumulating compatible solutes (Gunde‐Cimerman et al. 2018; Weinisch et al. 2018). We predict that related processes are involved in the increased salt tolerance in our experimental lines. The specific mutations underlying the adaptive responses will need to be further explored using genomic and functional studies, however, we predict that changes in gene copy number may play a role. The highly polyploid nature of the *Tetrahymena* genome provides an unusual opportunity for gene copy number variation via two distinct mechanisms: changes in chromosomal ploidy and gene duplication via somatic recombination. Both of these were found to be involved in adaptation to metal stress in *T. thermophila* (de Francisco et al. 2018). Changes in chromosome copy number, in particular, are interesting in the case of rapid environmental change because they are fast and reversible (de Francisco et al. 2018). Further studies exploring this connection between ciliate genome structure and response to rapid environmental change is of great interest.

## Author Contributions

**Rebecca A. Zufall:** conceptualization (equal), formal analysis (lead), funding acquisition (lead), supervision (equal), writing – original draft (lead). **Nia Pereda:** investigation (supporting), writing – review and editing (equal). **Karissa Plum:** investigation (supporting), supervision (equal), writing – review and editing (equal). **Ethan Rothschild:** conceptualization (equal), investigation (lead), writing – original draft (supporting).

## Funding

This work was supported by Division of Environmental Biology (2342961).

## Conflicts of Interest

The authors declare no conflicts of interest.

## Supporting information


**Table S1:** Survival comparisons among experimental conditions in different salt concentrations. Pairwise contrasts of marginal means of survival for experimental condition within each assay condition. Marginal means and contrasts were estimated from a bias‐reduced generalized linear model.
**Table S2:** Growth parameter comparisons among experimental conditions in different salt concentrations. Pairwise marginal contrasts among experimental conditions within each assay condition. Estimates represent differences in marginal means from linear mixed‐effects models.

## Data Availability

All data are available on Dryad: https://doi.org/10.5061/dryad.vhhmgqp6v.
