# Supplementary material for: Rapid Adaptation to Road Salts in a Freshwater Microbial Eukaryote
Source: Ecol Evol. 2026 Feb 25;16(3):e73160. doi: 10.1002/ece3.73160 (PMC12936414; doi:10.1002/ece3.73160)
Supplement: Supplementary file 1 — Table S1: Survival comparisons among experimental conditions in different salt concentrations. Pairwise contrasts of marginal means of survival for experimental condition within each assay condition. Marginal means and contrasts were estimated from a bias‐reduced generalized linear model. Table S2: Growth parameter comparisons among experimental conditions in different salt concentrations. Pairwise marginal contrasts among experimental conditions within each assay condition. Estimates represent differences in marginal means from linear mixed‐effects models. [file ECE3-16-e73160-s001.docx]

Supplemental Material

**Table S1. Survival comparisons among experimental conditions in different salt concentrations.** Pairwise contrasts of marginal means of survival for experimental condition within each assay condition. Marginal means and contrasts were estimated from a bias-reduced generalized linear model.

| Level 1 | Level 2 | Assay condition | Difference | SE | 95% CI | z | p-value |
| --- | --- | --- | --- | --- | --- | --- | --- |
| Co | An | SSP | 0.03 | 0.12 | -0.20, 0.27 | 0.28 | 0.782 |
| M | An | SSP | 0.03 | 0.12 | -0.20, 0.27 | 0.28 | 0.782 |
| M+ | An | SSP | -0.20 | 0.17 | -0.52, 0.13 | -1.19 | 0.233 |
| N | An | SSP | 0.03 | 0.12 | -0.20, 0.27 | 0.28 | 0.782 |
| N+ | An | SSP | 0.03 | 0.12 | -0.20, 0.27 | 0.28 | 0.782 |
| M | Co | SSP | 0.00 | 0.08 | -0.15, 0.15 | 0.00 | >0.999 |
| M+ | Co | SSP | -0.23 | 0.14 | -0.50, 0.04 | -1.65 | 0.098 |
| N | Co | SSP | 0.00 | 0.08 | -0.15, 0.15 | 0.00 | >0.999 |
| N+ | Co | SSP | 0.00 | 0.08 | -0.15, 0.15 | 0.00 | >0.999 |
| M+ | M | SSP | -0.23 | 0.14 | -0.50, 0.04 | -1.65 | 0.098 |
| N | M | SSP | 0.00 | 0.08 | -0.15, 0.15 | 0.00 | >0.999 |
| N+ | M | SSP | 0.00 | 0.08 | -0.15, 0.15 | 0.00 | >0.999 |
| N | M+ | SSP | 0.23 | 0.14 | -0.04, 0.50 | 1.65 | 0.098 |
| N+ | M+ | SSP | 0.23 | 0.14 | -0.04, 0.50 | 1.65 | 0.098 |
| N+ | N | SSP | 0.00 | 0.08 | -0.15, 0.15 | 0.00 | >0.999 |
| Co | An | Na9 | 0.31 | 0.15 | 0.02, 0.60 | 2.08 | 0.038 |
| M | An | Na9 | 0.31 | 0.15 | 0.02, 0.60 | 2.08 | 0.038 |
| M+ | An | Na9 | 0.31 | 0.15 | 0.02, 0.60 | 2.08 | 0.038 |
| N | An | Na9 | 0.31 | 0.15 | 0.02, 0.60 | 2.08 | 0.038 |
| N+ | An | Na9 | 0.31 | 0.15 | 0.02, 0.60 | 2.08 | 0.038 |
| M | Co | Na9 | 0.00 | 0.08 | -0.15, 0.15 | 0.00 | >0.999 |
| M+ | Co | Na9 | 0.00 | 0.08 | -0.15, 0.15 | 0.00 | >0.999 |
| N | Co | Na9 | 0.00 | 0.08 | -0.15, 0.15 | 0.00 | >0.999 |
| N+ | Co | Na9 | 0.00 | 0.08 | -0.15, 0.15 | 0.00 | >0.999 |
| M+ | M | Na9 | 0.00 | 0.08 | -0.15, 0.15 | 0.00 | >0.999 |
| N | M | Na9 | 0.00 | 0.08 | -0.15, 0.15 | 0.00 | >0.999 |
| N+ | M | Na9 | 0.00 | 0.08 | -0.15, 0.15 | 0.00 | >0.999 |
| N | M+ | Na9 | 0.00 | 0.08 | -0.15, 0.15 | 0.00 | >0.999 |
| N+ | M+ | Na9 | 0.00 | 0.08 | -0.15, 0.15 | 0.00 | >0.999 |
| N+ | N | Na9 | 0.00 | 0.08 | -0.15, 0.15 | 0.00 | >0.999 |
| Co | An | Na18 | 0.00 | 0.08 | -0.15, 0.15 | 0.00 | >0.999 |
| M | An | Na18 | 0.67 | 0.15 | 0.38, 0.96 | 4.53 | <0.001 |
| M+ | An | Na18 | 0.92 | 0.08 | 0.77, 1.08 | 11.76 | <0.001 |
| N | An | Na18 | 0.00 | 0.08 | -0.15, 0.15 | 0.00 | >0.999 |
| N+ | An | Na18 | 0.77 | 0.13 | 0.52, 1.02 | 6.08 | <0.001 |
| M | Co | Na18 | 0.67 | 0.15 | 0.38, 0.96 | 4.53 | <0.001 |
| M+ | Co | Na18 | 0.92 | 0.08 | 0.77, 1.08 | 11.76 | <0.001 |
| N | Co | Na18 | 0.00 | 0.08 | -0.15, 0.15 | 0.00 | >0.999 |
| N+ | Co | Na18 | 0.77 | 0.13 | 0.52, 1.02 | 6.08 | <0.001 |
| M+ | M | Na18 | 0.25 | 0.15 | -0.04, 0.54 | 1.71 | 0.087 |
| N | M | Na18 | -0.67 | 0.15 | -0.96, -0.38 | -4.53 | <0.001 |
| N+ | M | Na18 | 0.10 | 0.18 | -0.25, 0.45 | 0.56 | 0.577 |
| N | M+ | Na18 | -0.92 | 0.08 | -1.08, -0.77 | -11.76 | <0.001 |
| N+ | M+ | Na18 | -0.15 | 0.13 | -0.40, 0.09 | -1.22 | 0.224 |
| N+ | N | Na18 | 0.77 | 0.13 | 0.52, 1.02 | 6.08 | <0.001 |
| Co | An | Mg5 | -0.12 | 0.15 | -0.42, 0.18 | -0.78 | 0.435 |
| M | An | Mg5 | 0.03 | 0.12 | -0.20, 0.27 | 0.28 | 0.782 |
| M+ | An | Mg5 | 0.02 | 0.13 | -0.23, 0.27 | 0.17 | 0.867 |
| N | An | Mg5 | 0.03 | 0.12 | -0.20, 0.27 | 0.28 | 0.782 |
| N+ | An | Mg5 | 0.03 | 0.12 | -0.20, 0.27 | 0.28 | 0.782 |
| M | Co | Mg5 | 0.15 | 0.13 | -0.09, 0.40 | 1.22 | 0.224 |
| M+ | Co | Mg5 | 0.14 | 0.13 | -0.12, 0.41 | 1.05 | 0.292 |
| N | Co | Mg5 | 0.15 | 0.13 | -0.09, 0.40 | 1.22 | 0.224 |
| N+ | Co | Mg5 | 0.15 | 0.13 | -0.09, 0.40 | 1.22 | 0.224 |
| M+ | M | Mg5 | -0.01 | 0.09 | -0.19, 0.17 | -0.13 | 0.900 |
| N | M | Mg5 | 0.00 | 0.08 | -0.15, 0.15 | 0.00 | >0.999 |
| N+ | M | Mg5 | 0.00 | 0.08 | -0.15, 0.15 | 0.00 | >0.999 |
| N | M+ | Mg5 | 0.01 | 0.09 | -0.17, 0.19 | 0.13 | 0.900 |
| N+ | M+ | Mg5 | 0.01 | 0.09 | -0.17, 0.19 | 0.13 | 0.900 |
| N+ | N | Mg5 | 0.00 | 0.08 | -0.15, 0.15 | 0.00 | >0.999 |
| Co | An | Mg17 | -0.03 | 0.12 | -0.27, 0.20 | -0.28 | 0.782 |
| M | An | Mg17 | -0.03 | 0.12 | -0.27, 0.20 | -0.28 | 0.782 |
| M+ | An | Mg17 | 0.58 | 0.19 | 0.20, 0.95 | 3.04 | 0.002 |
| N | An | Mg17 | -0.03 | 0.12 | -0.27, 0.20 | -0.28 | 0.782 |
| N+ | An | Mg17 | 0.20 | 0.17 | -0.13, 0.52 | 1.19 | 0.233 |
| M | Co | Mg17 | 0.00 | 0.08 | -0.15, 0.15 | 0.00 | >0.999 |
| M+ | Co | Mg17 | 0.61 | 0.17 | 0.28, 0.94 | 3.63 | <0.001 |
| N | Co | Mg17 | 0.00 | 0.08 | -0.15, 0.15 | 0.00 | >0.999 |
| N+ | Co | Mg17 | 0.23 | 0.14 | -0.04, 0.50 | 1.65 | 0.098 |
| M+ | M | Mg17 | 0.61 | 0.17 | 0.28, 0.94 | 3.63 | <0.001 |
| N | M | Mg17 | 0.00 | 0.08 | -0.15, 0.15 | 0.00 | >0.999 |
| N+ | M | Mg17 | 0.23 | 0.14 | -0.04, 0.50 | 1.65 | 0.098 |
| N | M+ | Mg17 | -0.61 | 0.17 | -0.94, -0.28 | -3.63 | <0.001 |
| N+ | M+ | Mg17 | -0.38 | 0.20 | -0.78, 0.02 | -1.87 | 0.062 |
| N+ | N | Mg17 | 0.23 | 0.14 | -0.04, 0.50 | 1.65 | 0.098 |

**Table S2.** **Growth parameter comparisons among experimental conditions in different salt concentrations.** Pairwise marginal contrasts among experimental conditions within each assay condition. Estimates represent differences in marginal means from linear mixed-effects models.

**Lag time:** Assay condition **SSP**

| Contrast | Estimate | SE | df | t-ratio | p-value |
| --- | --- | --- | --- | --- | --- |
| An - Co | 6.05 | 3.51 | 38.6 | 1.726 | 0.5236 |
| An - M | 1.66 | 3.51 | 38.6 | 0.473 | 0.9968 |
| An - (M+) | 0.21 | 3.68 | 42.9 | 0.057 | 1.0 |
| An - N | -7.62 | 3.51 | 38.6 | -2.172 | 0.2738 |
| An - (N+) | -1.64 | 3.51 | 38.6 | -0.468 | 0.997 |
| Co - M | -4.4 | 2.72 | 77.5 | -1.614 | 0.5918 |
| Co - (M+) | -5.84 | 2.94 | 85.2 | -1.987 | 0.3581 |
| Co - N | -13.67 | 2.72 | 77.5 | -5.022 | <.0001 |
| Co - (N+) | -7.69 | 2.72 | 77.5 | -2.826 | 0.0639 |
| M - (M+) | -1.45 | 2.94 | 85.2 | -0.493 | 0.9963 |
| M - N | -9.28 | 2.72 | 77.5 | -3.407 | 0.0129 |
| M - (N+) | -3.3 | 2.72 | 77.5 | -1.212 | 0.83 |
| (M+) - N | -7.83 | 2.94 | 85.2 | -2.662 | 0.0939 |
| (M+) - (N+) | -1.85 | 2.94 | 85.2 | -0.629 | 0.9885 |
| N - (N+) | 5.98 | 2.72 | 77.5 | 2.196 | 0.2516 |

**Lag time:** Assay condition **Na9**

| Contrast | Estimate | SE | df | t-ratio | p-value |
| --- | --- | --- | --- | --- | --- |
| An - Co | 49.68 | 3.28 | 29.9 | 15.159 | <.0001 |
| An - M | 52.21 | 3.28 | 29.9 | 15.929 | <.0001 |
| An - (M+) | 34.74 | 3.28 | 29.9 | 10.601 | <.0001 |
| An - N | 48.3 | 3.28 | 29.9 | 14.738 | <.0001 |
| An - (N+) | 35.98 | 3.28 | 29.9 | 10.977 | <.0001 |
| Co - M | 2.52 | 2.72 | 77.5 | 0.927 | 0.9385 |
| Co - (M+) | -14.94 | 2.72 | 77.5 | -5.487 | <.0001 |
| Co - N | -1.38 | 2.72 | 77.5 | -0.507 | 0.9958 |
| Co - (N+) | -13.71 | 2.72 | 77.5 | -5.034 | <.0001 |
| M - (M+) | -17.46 | 2.72 | 77.5 | -6.413 | <.0001 |
| M - N | -3.9 | 2.72 | 77.5 | -1.434 | 0.7064 |
| M - (N+) | -16.23 | 2.72 | 77.5 | -5.961 | <.0001 |
| (M+) - N | 13.56 | 2.72 | 77.5 | 4.979 | 0.0001 |
| (M+) - (N+) | 1.23 | 2.72 | 77.5 | 0.452 | 0.9975 |
| N - (N+) | -12.33 | 2.72 | 77.5 | -4.527 | 0.0003 |

**Lag time:** Assay condition **Na18**

| Contrast | Estimate | SE | df | t-ratio | p-value |
| --- | --- | --- | --- | --- | --- |
| An - Co | nonEst | NA | NA | NA | NA |
| An - M | nonEst | NA | NA | NA | NA |
| An - (M+) | nonEst | NA | NA | NA | NA |
| An - N | nonEst | NA | NA | NA | NA |
| An - (N+) | nonEst | NA | NA | NA | NA |
| Co - M | nonEst | NA | NA | NA | NA |
| Co - (M+) | nonEst | NA | NA | NA | NA |
| Co - N | nonEst | NA | NA | NA | NA |
| Co - (N+) | nonEst | NA | NA | NA | NA |
| M - (M+) | 14.98 | 3.03 | 94.1 | 4.947 | <.0001 |
| M - N | nonEst | NA | NA | NA | NA |
| M - (N+) | -14.4 | 3.13 | 102.1 | -4.596 | <.0001 |
| (M+) - N | nonEst | NA | NA | NA | NA |
| (M+) - (N+) | -29.38 | 2.84 | 86.7 | -10.352 | <.0001 |
| N - (N+) | nonEst | NA | NA | NA | NA |

**Lag time:** Assay condition **Mg5**

| Contrast | Estimate | SE | df | t-ratio | p-value |
| --- | --- | --- | --- | --- | --- |
| An - Co | 10.5 | 3.6 | 42.1 | 2.918 | 0.0587 |
| An - M | -3.72 | 3.51 | 38.6 | -1.062 | 0.8934 |
| An - (M+) | -1.37 | 3.68 | 42.9 | -0.373 | 0.999 |
| An - N | 1.38 | 3.51 | 38.6 | 0.393 | 0.9987 |
| An - (N+) | -29.77 | 3.51 | 38.6 | -8.489 | <.0001 |
| Co - M | -14.22 | 2.84 | 86.5 | -5.01 | <.0001 |
| Co - (M+) | -11.87 | 3.05 | 93.3 | -3.894 | 0.0025 |
| Co - N | -9.12 | 2.84 | 86.5 | -3.212 | 0.0221 |
| Co - (N+) | -40.27 | 2.84 | 86.5 | -14.186 | <.0001 |
| M - (M+) | 2.35 | 2.94 | 85.2 | 0.8 | 0.9668 |
| M - N | 5.1 | 2.72 | 77.5 | 1.874 | 0.4258 |
| M - (N+) | -26.05 | 2.72 | 77.5 | -9.568 | <.0001 |
| (M+) - N | 2.75 | 2.94 | 85.2 | 0.935 | 0.9362 |
| (M+) - (N+) | -28.4 | 2.94 | 85.2 | -9.658 | <.0001 |
| N - (N+) | -31.15 | 2.72 | 77.5 | -11.442 | <.0001 |

**Lag time:** Assay condition **Mg17**

| Contrast | Estimate | SE | df | t-ratio | p-value |
| --- | --- | --- | --- | --- | --- |
| An - Co | nonEst | NA | NA | NA | NA |
| An - M | nonEst | NA | NA | NA | NA |
| An - (M+) | nonEst | NA | NA | NA | NA |
| An - N | nonEst | NA | NA | NA | NA |
| An - (N+) | nonEst | NA | NA | NA | NA |
| Co - M | nonEst | NA | NA | NA | NA |
| Co - (M+) | nonEst | NA | NA | NA | NA |
| Co - N | nonEst | NA | NA | NA | NA |
| Co - (N+) | nonEst | NA | NA | NA | NA |
| M - (M+) | nonEst | NA | NA | NA | NA |
| M - N | nonEst | NA | NA | NA | NA |
| M - (N+) | nonEst | NA | NA | NA | NA |
| (M+) - N | nonEst | NA | NA | NA | NA |
| (M+) - (N+) | 25.22 | 4.65 | 151.2 | 5.427 | <.0001 |
| N - (N+) | nonEst | NA | NA | NA | NA |

**Growth rate:** Assay condition **SSP**

| Contrast | Estimate | SE | df | t-ratio | p-value |
| --- | --- | --- | --- | --- | --- |
| An - Co | 0.0114 | 0.0108 | 35.8 | 1.054 | 0.8961 |
| An - M | -0.0134 | 0.0108 | 35.8 | -1.239 | 0.8147 |
| An - (M+) | 0.0204 | 0.0113 | 39.8 | 1.803 | 0.4752 |
| An - N | 0.0213 | 0.0108 | 35.8 | 1.976 | 0.3754 |
| An - (N+) | 0.0483 | 0.0108 | 35.8 | 4.469 | 0.001 |
| Co - M | -0.0248 | 0.00836 | 71.9 | -2.962 | 0.0458 |
| Co - (M+) | 0.00904 | 0.00903 | 79.4 | 1.002 | 0.916 |
| Co - N | 0.00996 | 0.00836 | 71.9 | 1.192 | 0.8394 |
| Co - (N+) | 0.0369 | 0.00836 | 71.9 | 4.413 | 0.0005 |
| M - (M+) | 0.0338 | 0.00903 | 79.4 | 3.745 | 0.0044 |
| M - N | 0.0347 | 0.00836 | 71.9 | 4.154 | 0.0012 |
| M - (N+) | 0.0617 | 0.00836 | 71.9 | 7.375 | <.0001 |
| (M+) - N | 0.000919 | 0.00903 | 79.4 | 0.102 | 1.0 |
| (M+) - (N+) | 0.0278 | 0.00903 | 79.4 | 3.085 | 0.0323 |
| N - (N+) | 0.0269 | 0.00836 | 71.9 | 3.221 | 0.0227 |

**Growth rate:** Assay condition **Na9**

| Contrast | Estimate | SE | df | t-ratio | p-value |
| --- | --- | --- | --- | --- | --- |
| An - Co | 0.0794 | 0.0101 | 27.9 | 7.857 | <.0001 |
| An - M | 0.0794 | 0.0101 | 27.9 | 7.858 | <.0001 |
| An - (M+) | 0.0468 | 0.0102 | 29.2 | 4.568 | 0.0011 |
| An - N | 0.0756 | 0.0101 | 27.9 | 7.485 | <.0001 |
| An - (N+) | 0.0444 | 0.0101 | 27.9 | 4.393 | 0.0018 |
| Co - M | 3.63e-06 | 0.00836 | 71.9 | 0.0 | 1.0 |
| Co - (M+) | -0.0326 | 0.00852 | 75.8 | -3.827 | 0.0035 |
| Co - N | -0.00376 | 0.00836 | 71.9 | -0.45 | 0.9976 |
| Co - (N+) | -0.035 | 0.00836 | 71.9 | -4.189 | 0.0011 |
| M - (M+) | -0.0326 | 0.00852 | 75.8 | -3.828 | 0.0035 |
| M - N | -0.00377 | 0.00836 | 71.9 | -0.451 | 0.9976 |
| M - (N+) | -0.035 | 0.00836 | 71.9 | -4.189 | 0.0011 |
| (M+) - N | 0.0289 | 0.00852 | 75.8 | 3.386 | 0.0138 |
| (M+) - (N+) | -0.00239 | 0.00852 | 75.8 | -0.281 | 0.9998 |
| N - (N+) | -0.0312 | 0.00836 | 71.9 | -3.738 | 0.0048 |

**Growth rate:** Assay condition **Na18**

| Contrast | Estimate | SE | df | t-ratio | p-value |
| --- | --- | --- | --- | --- | --- |
| An - Co | nonEst | NA | NA | NA | NA |
| An - M | nonEst | NA | NA | NA | NA |
| An - (M+) | nonEst | NA | NA | NA | NA |
| An - N | nonEst | NA | NA | NA | NA |
| An - (N+) | nonEst | NA | NA | NA | NA |
| Co - M | nonEst | NA | NA | NA | NA |
| Co - (M+) | nonEst | NA | NA | NA | NA |
| Co - N | nonEst | NA | NA | NA | NA |
| Co - (N+) | nonEst | NA | NA | NA | NA |
| M - (M+) | -0.0294 | 0.00929 | 87.9 | -3.161 | 0.0061 |
| M - N | nonEst | NA | NA | NA | NA |
| M - (N+) | -0.0884 | 0.00961 | 95.4 | -9.203 | <.0001 |
| (M+) - N | nonEst | NA | NA | NA | NA |
| (M+) - (N+) | -0.0591 | 0.00871 | 80.4 | -6.781 | <.0001 |
| N - (N+) | nonEst | NA | NA | NA | NA |

**Growth rate:** Assay condition **Mg5**

| Contrast | Estimate | SE | df | t-ratio | p-value |
| --- | --- | --- | --- | --- | --- |
| An - Co | 0.0408 | 0.0111 | 39.0 | 3.683 | 0.0085 |
| An - M | -0.0222 | 0.0108 | 35.8 | -2.054 | 0.3338 |
| An - (M+) | -0.00988 | 0.0113 | 39.8 | -0.872 | 0.951 |
| An - N | 0.0133 | 0.0108 | 35.8 | 1.231 | 0.8189 |
| An - (N+) | -0.0979 | 0.0127 | 57.1 | -7.713 | <.0001 |
| Co - M | -0.063 | 0.00871 | 80.3 | -7.227 | <.0001 |
| Co - (M+) | -0.0507 | 0.00935 | 87.0 | -5.416 | <.0001 |
| Co - N | -0.0275 | 0.00871 | 80.3 | -3.156 | 0.0264 |
| Co - (N+) | -0.139 | 0.011 | 121.2 | -12.639 | <.0001 |
| M - (M+) | 0.0123 | 0.00903 | 79.4 | 1.363 | 0.7489 |
| M - N | 0.0355 | 0.00836 | 71.9 | 4.243 | 0.0009 |
| M - (N+) | -0.0757 | 0.0107 | 115.5 | -7.081 | <.0001 |
| (M+) - N | 0.0232 | 0.00903 | 79.4 | 2.567 | 0.1178 |
| (M+) - (N+) | -0.088 | 0.0112 | 118.2 | -7.843 | <.0001 |
| N - (N+) | -0.111 | 0.0107 | 115.5 | -10.397 | <.0001 |

**Growth rate:** Assay condition **Mg17**

| Contrast | Estimate | SE | df | t-ratio | p-value |
| --- | --- | --- | --- | --- | --- |
| An - Co | nonEst | NA | NA | NA | NA |
| An - M | nonEst | NA | NA | NA | NA |
| An - (M+) | nonEst | NA | NA | NA | NA |
| An - N | nonEst | NA | NA | NA | NA |
| An - (N+) | nonEst | NA | NA | NA | NA |
| Co - M | nonEst | NA | NA | NA | NA |
| Co - (M+) | nonEst | NA | NA | NA | NA |
| Co - N | nonEst | NA | NA | NA | NA |
| Co - (N+) | nonEst | NA | NA | NA | NA |
| M - (M+) | nonEst | NA | NA | NA | NA |
| M - N | nonEst | NA | NA | NA | NA |
| M - (N+) | nonEst | NA | NA | NA | NA |
| (M+) - N | nonEst | NA | NA | NA | NA |
| (M+) - (N+) | -0.00992 | 0.0143 | 139.2 | -0.695 | 0.4882 |
| N - (N+) | nonEst | NA | NA | NA | NA |

**Carrying capacity:** Assay condition **SSP**

| Contrast | Estimate | SE | df | t-ratio | p-value |
| --- | --- | --- | --- | --- | --- |
| An - Co | 0.02313 | 0.0259 | 23.2 | 0.894 | 0.944 |
| An - M | 0.05531 | 0.0259 | 23.2 | 2.138 | 0.3034 |
| An - (M+) | -0.02119 | 0.0267 | 25.6 | -0.793 | 0.9662 |
| An - N | 0.0015 | 0.0259 | 23.2 | 0.058 | 1.0 |
| An - (N+) | 0.17684 | 0.0259 | 23.2 | 6.837 | <.0001 |
| Co - M | 0.03217 | 0.0187 | 38.1 | 1.724 | 0.5252 |
| Co - (M+) | -0.04433 | 0.0198 | 44.9 | -2.235 | 0.2425 |
| Co - N | -0.02163 | 0.0187 | 38.1 | -1.159 | 0.8531 |
| Co - (N+) | 0.15371 | 0.0187 | 38.1 | 8.236 | <.0001 |
| M - (M+) | -0.0765 | 0.0198 | 44.9 | -3.857 | 0.0046 |
| M - N | -0.0538 | 0.0187 | 38.1 | -2.883 | 0.0657 |
| M - (N+) | 0.12154 | 0.0187 | 38.1 | 6.512 | <.0001 |
| (M+) - N | 0.0227 | 0.0198 | 44.9 | 1.144 | 0.86 |
| (M+) - (N+) | 0.19804 | 0.0198 | 44.9 | 9.985 | <.0001 |
| N - (N+) | 0.17534 | 0.0187 | 38.1 | 9.395 | <.0001 |

**Carrying capacity:** Assay condition **Na9**

| Contrast | Estimate | SE | df | t-ratio | p-value |
| --- | --- | --- | --- | --- | --- |
| An - Co | -0.20936 | 0.0256 | 22.1 | -8.168 | <.0001 |
| An - M | -0.28483 | 0.0256 | 22.1 | -11.118 | <.0001 |
| An - (M+) | -0.13818 | 0.0249 | 19.9 | -5.556 | 0.0003 |
| An - N | -0.22495 | 0.0249 | 19.9 | -9.044 | <.0001 |
| An - (N+) | -0.09594 | 0.0249 | 19.9 | -3.857 | 0.011 |
| Co - M | -0.07547 | 0.0206 | 52.0 | -3.664 | 0.0073 |
| Co - (M+) | 0.07118 | 0.0197 | 45.0 | 3.62 | 0.0091 |
| Co - N | -0.01559 | 0.0197 | 45.0 | -0.793 | 0.9673 |
| Co - (N+) | 0.11342 | 0.0197 | 45.0 | 5.768 | <.0001 |
| M - (M+) | 0.14664 | 0.0196 | 45.1 | 7.464 | <.0001 |
| M - N | 0.05988 | 0.0196 | 45.1 | 3.048 | 0.0419 |
| M - (N+) | 0.18888 | 0.0196 | 45.1 | 9.614 | <.0001 |
| (M+) - N | -0.08677 | 0.0187 | 38.1 | -4.649 | 0.0005 |
| (M+) - (N+) | 0.04224 | 0.0187 | 38.1 | 2.263 | 0.234 |
| N - (N+) | 0.12901 | 0.0187 | 38.1 | 6.912 | <.0001 |

**Carrying capacity:** Assay condition **Na18**

| Contrast | Estimate | SE | df | t-ratio | p-value |
| --- | --- | --- | --- | --- | --- |
| An - Co | nonEst | NA | NA | NA | NA |
| An - M | nonEst | NA | NA | NA | NA |
| An - (M+) | nonEst | NA | NA | NA | NA |
| An - N | nonEst | NA | NA | NA | NA |
| An - (N+) | nonEst | NA | NA | NA | NA |
| Co - M | nonEst | NA | NA | NA | NA |
| Co - (M+) | nonEst | NA | NA | NA | NA |
| Co - N | nonEst | NA | NA | NA | NA |
| Co - (N+) | nonEst | NA | NA | NA | NA |
| M - (M+) | 0.00718 | 0.0306 | 126.0 | 0.235 | 0.97 |
| M - N | nonEst | NA | NA | NA | NA |
| M - (N+) | 0.1508 | 0.0311 | 129.9 | 4.842 | <.0001 |
| (M+) - N | nonEst | NA | NA | NA | NA |
| (M+) - (N+) | 0.14362 | 0.0196 | 45.2 | 7.319 | <.0001 |
| N - (N+) | nonEst | NA | NA | NA | NA |

**Carrying capacity:** Assay condition **Mg5**

| Contrast | Estimate | SE | df | t-ratio | p-value |
| --- | --- | --- | --- | --- | --- |
| An - Co | 0.0068 | 0.0266 | 25.5 | 0.256 | 0.9998 |
| An - M | 0.05844 | 0.0259 | 23.2 | 2.259 | 0.2503 |
| An - (M+) | 0.00169 | 0.0267 | 25.6 | 0.063 | 1.0 |
| An - N | 0.03279 | 0.0259 | 23.2 | 1.268 | 0.7989 |
| An - (N+) | 0.14782 | 0.0259 | 23.2 | 5.715 | 0.0001 |
| Co - M | 0.05164 | 0.0197 | 45.0 | 2.626 | 0.1118 |
| Co - (M+) | -0.00511 | 0.0208 | 51.7 | -0.246 | 0.9999 |
| Co - N | 0.02599 | 0.0197 | 45.0 | 1.322 | 0.7714 |
| Co - (N+) | 0.14102 | 0.0197 | 45.0 | 7.172 | <.0001 |
| M - (M+) | -0.05675 | 0.0198 | 44.9 | -2.861 | 0.0658 |
| M - N | -0.02564 | 0.0187 | 38.1 | -1.374 | 0.7419 |
| M - (N+) | 0.08938 | 0.0187 | 38.1 | 4.789 | 0.0003 |
| (M+) - N | 0.03111 | 0.0198 | 44.9 | 1.568 | 0.623 |
| (M+) - (N+) | 0.14613 | 0.0198 | 44.9 | 7.368 | <.0001 |
| N - (N+) | 0.11503 | 0.0187 | 38.1 | 6.163 | <.0001 |

**Carrying capacity:** Assay condition **Mg17**

| Contrast | Estimate | SE | df | t-ratio | p-value |
| --- | --- | --- | --- | --- | --- |
| An - Co | nonEst | NA | NA | NA | NA |
| An - M | nonEst | NA | NA | NA | NA |
| An - (M+) | nonEst | NA | NA | NA | NA |
| An - N | nonEst | NA | NA | NA | NA |
| An - (N+) | nonEst | NA | NA | NA | NA |
| Co - M | nonEst | NA | NA | NA | NA |
| Co - (M+) | nonEst | NA | NA | NA | NA |
| Co - N | nonEst | NA | NA | NA | NA |
| Co - (N+) | nonEst | NA | NA | NA | NA |
| M - (M+) | nonEst | NA | NA | NA | NA |
| M - N | nonEst | NA | NA | NA | NA |
| M - (N+) | nonEst | NA | NA | NA | NA |
| (M+) - N | nonEst | NA | NA | NA | NA |
| (M+) - (N+) | nonEst | NA | NA | NA | NA |
| N - (N+) | nonEst | NA | NA | NA | NA |
